# Controlled Phase Changes of Titania Using Nitrogen Plasma

**DOI:** 10.1186/s11671-016-1821-y

**Published:** 2017-01-13

**Authors:** R. Trejo-Tzab, Liliana Caballero-Espada, P. Quintana, Alejandro Ávila-Ortega, R. A. Medina-Esquivel

**Affiliations:** 1 Facultad de Ingeniería Química, Universidad Autónoma de Yucatán, Mérida, Yucatán 97203 México; 2 Depto. de Física Aplicada, CINVESTAV-Unidad Mérida, A.P. 73, Cordemex, 97310 Mérida, Yucatán México; 3 Facultad de Ingeniería, Universidad Autónoma de Yucatán, Mérida, Yucatán 97203 México

**Keywords:** Nitrogen plasma, Plasma treatment, Plasma discharge, Titania

## Abstract

In this work, the development of a new crystallization technique is reported, using nitrogen plasma (AC) to obtain nanostructured anatase and rutile from amorphous titanium oxide (TiO_2_). This methodology increases throughput and minimizes thermal effects. Nanostructured amorphous TiO_2_ was obtained by the sol-gel method and subsequently subjected to AC treatment, at a controlled pressure, applying different powers and treatment times in order to obtain phase changes. The obtained samples were characterized using X-ray diffraction (XRD), thermogravimetric analysis (TGA), and X-ray photoelectron spectroscopy (XPS). The results show the crystallization in parallel with anatase and rutile phases with a proportion that is directly related to the applied power in the plasma and the treatment time. This technique allows us to obtain smaller crystals in comparison with those of classic thermal methodologies. It is also demonstrated that the application of plasma represents a novel and innovative method to obtain phase polymorphic changes in titanium oxide without needing to apply prolonged heat treatments at high temperatures and can therefore be taken into consideration as a technique with low energy costs, in comparison with conventional heat treatments.

## Background

Titanium oxide (TiO_2_) is a semiconductor material which has been widely investigated due to its electrical and optical properties. It presents good chemical stability, resistance to corrosion, and considerable oxidizing power [1–6]. Because of these properties, TiO_2_ is applied in environmental purification [7], hydrogen production [8], optoelectronics [9], and other applications [10, 11]. Moreover, titanium oxide has shown good results as a photocatalyst to be used in the degradation of pollutants in wastewater [12–14]. The diversity of applications, which TiO_2_ has shown to possess, as a result of the reactions takes place on its surface, due to the generation of electron-hole pairs [15]. However, in order for titanium oxide to present photoactivity, it must be in one of its three crystalline phases (anatase, rutile, or brookite); anatase being the most used in photocatalysis due to the low recombination of the photogenerated electron-hole pair [16, 17].

The wide range of applications of TiO_2_ has motivated diverse research groups to develop and optimize the sol-gel preparation method in order to obtain amorphous titanium oxide; nevertheless, irrespective of the sol-gel techniques that have been developed, the conventional method to obtain the crystalline phases, in particular anatase and rutile, has been carried out exclusively by thermal treatments. It is important to take into account the high power consumption used to reach the high temperatures that are required to obtain the phase changes and the long treatment times. Thus, there is a need to develop new methodologies which will simultaneously reduce power expenses in the phase transformations of TiO_2_ and to control the formation of polymorphic phases.

In previous studies [18, 19], we have reported the development of a new methodology in which argon and nitrogen plasma were used to impregnate metal nanoparticles on titanium oxide using metal foil as electrode. We have also reported the phase changes that could be presented by titanium oxide P25 when applying plasma on nanostructured titanium P25 powder and the incorporation of nitrogen in the crystal lattice of the TiO_2_ to obtain N-doped TiO_2-x_. These studies have tended to focus more on the impregnation of metals with plasma, where the power and treatment times did not exceed the established experimental limits, in such a way that the phase changes are not significant in the processes of metallic impregnation on titanium. Also, other researchers have confirmed the importance of incorporating nitrogen into TiO_2_ crystal lattice [20, 21].

Previous experiences have allowed us to implement the plasma method (nitrogen plasma (AC)) as a novel and innovative technique, which is both fast and simple, in order to achieve the crystallization of the amorphous TiO_2_ in order to obtain anatase and rutile phases with a minimum power cost, in comparison with the conventional heat treatments.

## Methods

### Synthesis of Amorphous TiO_2_ by Sol-Gel

A butoxide titanium solution 97% Ti[O(CH_2_)_3_]_4_CH_3_ was used as a precursor for the synthesis of amorphous TiO_2_ by means of the sol-gel method. First, the alcohol (1-butanol anhydrous C_4_H_10_O) was placed in a three-necked flask and maintained in vigorous agitation. Subsequently, butoxide was added to the mixture with a slow drip process, maintaining a constant temperature over a period of 24 h, after which the mixture was centrifuged at 7500 rpm for 15 min. Supernatant liquid was separated by decantation, and the material obtained was subjected to a drying process at 70 °C for 24 h and calcined at 260 °C for 1 h.

### Heat Treatments and Plasma Treatments

**Fig. 1:**
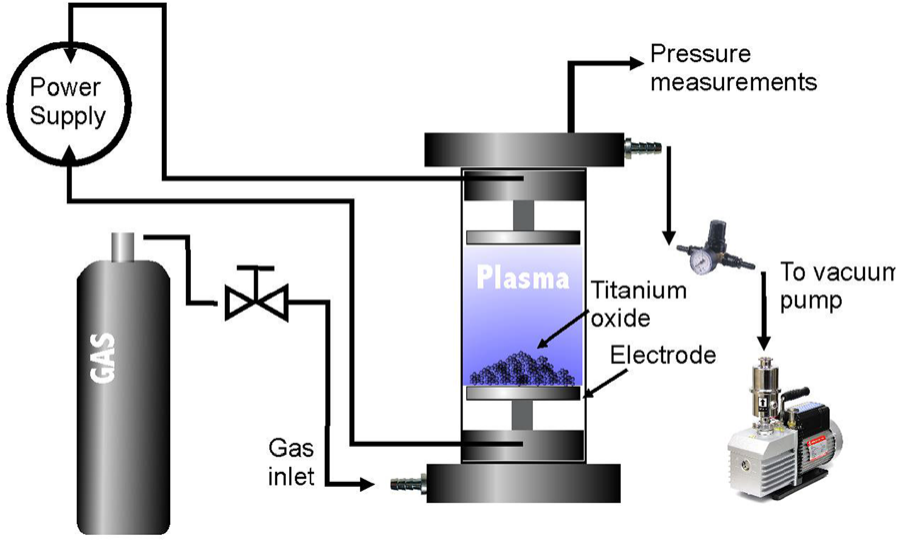
Schematic diagram of the experimental arrangement of the treatment

Amorphous titanium oxide samples were subjected to heat treatments of 500, 600, and 700 °C at periods of 1, 2, and 3 h, respectively, to induce the crystallization of TiO_2_. A variable power plasma reactor was used (0–250 W) to treat TiO_2_ samples in nitrogen plasma (Fig. 1). Throughout this study, the intensity of the power supply will be denoted in percentages, from 0% for 0 W up to 100% for 250 W. The samples of amorphous TiO_2_ were placed on one of the electrodes where discharge is carried out inside the plasma chamber. Diffusion pump (Edwards, Mod. E2M0.7) was used to obtain an optimal vacuum and uniform discharge of plasma on the amorphous samples of TiO_2_. After evacuating the reactor with the diffusion pump, was introduced N_2_ (99.99%) into the reactor plasma. The samples were then treated at different intensities of power (60–100%) and treatment times of 60 and 120 min, maintaining a flow of nitrogen gas inside the reactor at a constant pressure of ~30 Pa.

### Characterization

The crystalline structure of titanium oxide powder samples was analyzed using X-ray diffraction (XRD, BRUKER D8 ADVANCE) with Cu kα wavelength 1.5418 Å at 30 mA. The patterns were recorded in a range from 10° to 90° (2θ). The thermogravimetric analyses to determine the thermal properties were performed under nitrogen atmosphere with thermogravimetric analysis (TGA) Discovery Series equipment, applying a speed of 20 °C/min to increase the temperature from 25 to 800 °C. Surface composition and chemical state of the samples were also performed; X-ray photoelectron spectroscopy (XPS) was conducted on a Thermo Fisher Scientific model K-alpha with a source of the Kα (1400 eV).

## Results and Discussion

### TGA and XRD Analysis for Amorphous Samples

**Fig. 2:**
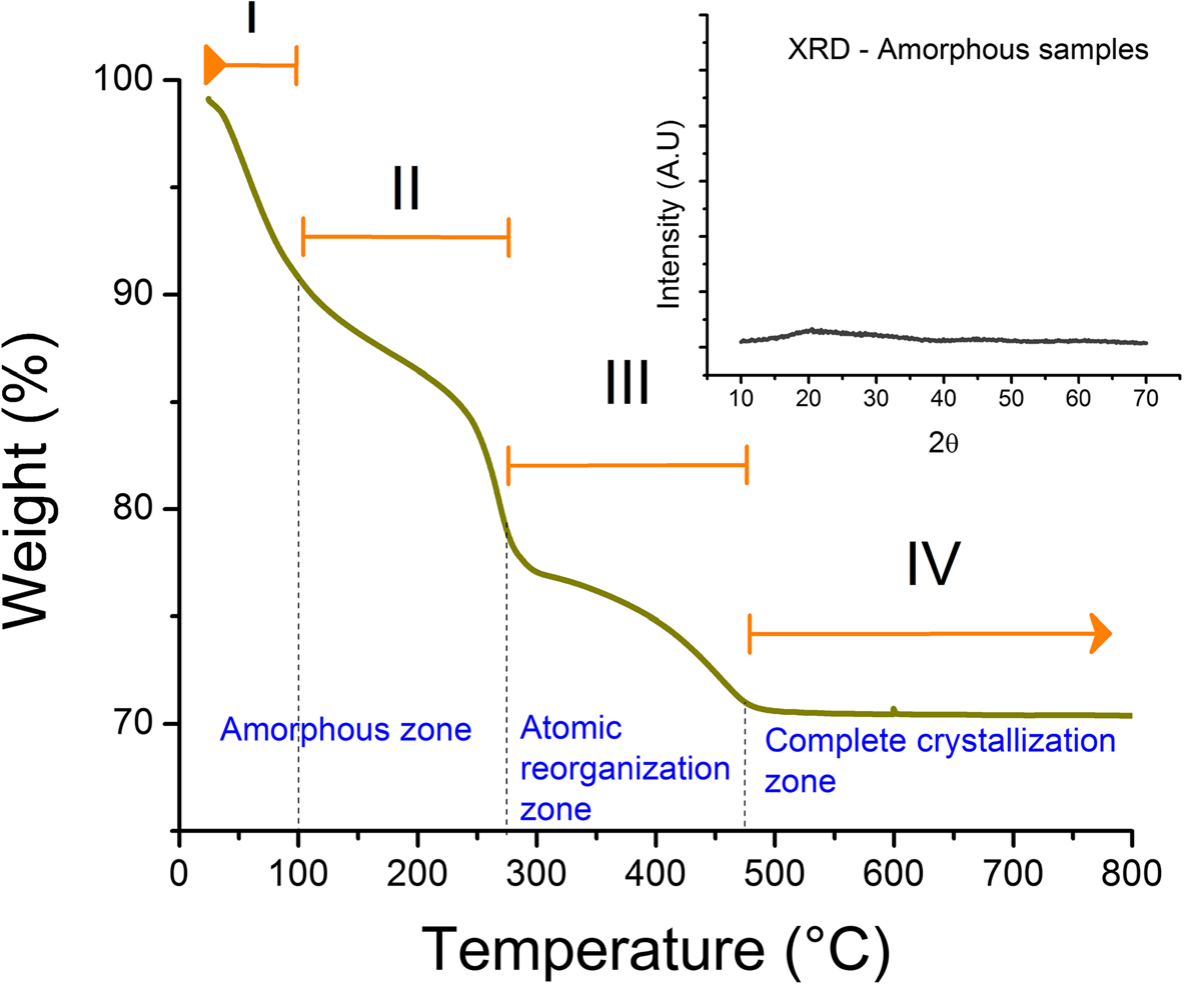
TGA and XRD of amorphous TiO_2_ synthesized by the sol-gel method

After the synthesis by the sol-gel method and before any thermal and plasma treatment, the obtained samples were characterized by XRD and TGA techniques (Fig. 2). In this case, XRD was used to determine the presence (or absence) of crystalline phases; the diffraction patterns (Fig. 2 (inset)) show the absence of any clearly characteristic peaks; therefore, the titanium oxide samples are in amorphous nature. On the other hand, the amorphous TiO_2_ samples contain impurities due to the synthesis process; the dynamic release of humidity and organic waste for TiO_2_ is a widely studied process from the thermogravimetric studies (TGA). Unlike other materials, the TiO_2_ presents polymorphic changes at elevated temperatures, initiating this process at 300 °C [22, 23] and presenting a complete phase transformation at a temperature above 500 °C. Based on the results obtained from this analysis, the amorphous TiO_2_ thermogram (Fig. 2) has been divided into four stages, grouped together in three zones: (a) amorphous zone: stages I and II; (b) atomic reorganization zone: stage III; and (c) complete crystallization zone: stage IV.

The amorphous zone initiates with stage I at 25 °C and ends on stage II at 100 °C, and it is characterized by water loss and the evaporation of solvents with a weight loss of ~10%. Stage II, with a temperature range of 100–265 °C, is associated with the combustion of organic material and a loss by hydroxyl desorption adsorbed in the sample, with an accumulated total weight loss of ~22%. We took 260 °C (5 °C before the limit temperature of this stage) as the thermal pretreatment temperature for the amorphous samples, before being treated with nitrogen plasma. Thermal pretreatment does not induce phase changes and allows obtaining amorphous TiO_2_ sample with 22% less impurities.

The atomic reorganization zone (stage III) initiates at a temperature of 265 °C and ends at 500 °C. This stage can be explained by taking into consideration the onset of phase changes undergone by titanium oxide as it passes from an amorphous state to its first crystallization phase, anatase [22], as was demonstrated later through XRD analyses carried out on TiO_2_ samples, after the series of heat treatments. In this transition zone, the amorphous TiO_2_ begins to undergo changes in its amorphous state giving rise to a crystalline structure (atomic reorganization) induced by high temperature. This atomic reorganization allows the desorption of the remaining organic residues in the internal part of the TiO_2_, lattice structure, which are released as soon as the internal structure has changed progressively and the total crystallization has been achieved, giving rise to a complete atomic order and a defined formation of the unit cell of the TiO_2_ lattice structure. This progressive change of the atomic ordering allows the liberation of chemical residues caught in the lattice structure.

The zone of complete crystallization corresponding to stage IV does not present weight loss. The titanium oxide is in its phase of anatase, rutile, or a mixture of both, which will depend on the temperature of the heat treatment. TGA analysis shows that, once this stage has been reached, the samples have experienced a total weight loss of ~30%, which does not vary throughout this last stage [23].

### Heat Treatments

**Fig. 3:**
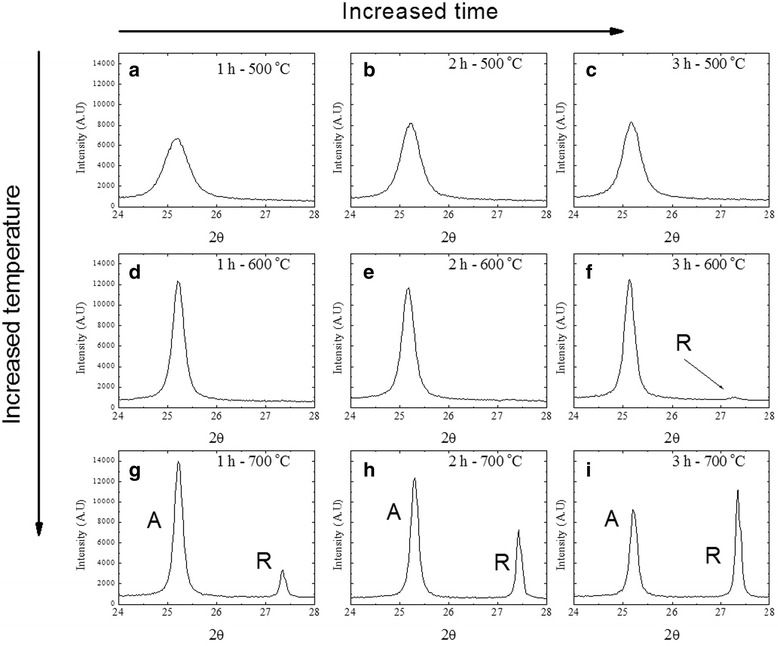
Diffractograms of amorphous TiO_2_ after the heat treatment at 500, 600, and 700 °C, in time intervals of 1, 2, and 3 h (**a**–**i**)

XRD analyses (Fig. 3) of titanium oxide samples treated thermally at 500, 600, and 700 °C in time intervals of 1, 2, and 3 h, respectively, reveal a selective crystallization. One can observe that the crystalline phase of anatase prevails when heat treatments of 500 and 600 °C with treatment times from 1 to 3 h are applied. Crystallization of TiO_2_ into its anatase phase can be observed through the evolution of the maximum peak signal corresponding to the plane (101) depending on the treatment time and maintaining a constant temperature; a significant change in intensity and width in the semiheight of the peak maximum of anatase can be appreciated; (Fig. 3a–f) for 500 and 600 °C, respectively. Also, the crystallization of rutile phase can also be observed with a heat treatment at 600 °C for 3 h (Fig. 3f).

More significant changes in the process of crystallization of TiO_2_ can be observed when the temperature is increased and the heat treatment time remains constant (Fig. 3 from top to bottom). An increase in the intensity and a significant reduction in the width of the semiheight of the anatase peak (101) can be observed. However, when the temperature increases up to 700 °C, besides changing the intensity and width of the semiheight of the peak maximum, it also gives rise to the formation of a second phase of the TiO_2_ corresponding to rutile, which can be observed at the peak maximum of rutile (110). At this temperature, a combination of both phases can be obtained. On the other hand, an increase of rutile and a decrease of anatase can be observed in Fig. 3g–i, which is in relation to a treatment time of 1–3 h.

XRD results reveal that the formation of anatase and rutile using the heat treatment is obtained in a series of processes, that is to say, beginning with the formation of anatase and following with the formation of rutile.

### Treatment with Nitrogen Plasma

**Fig. 4:**
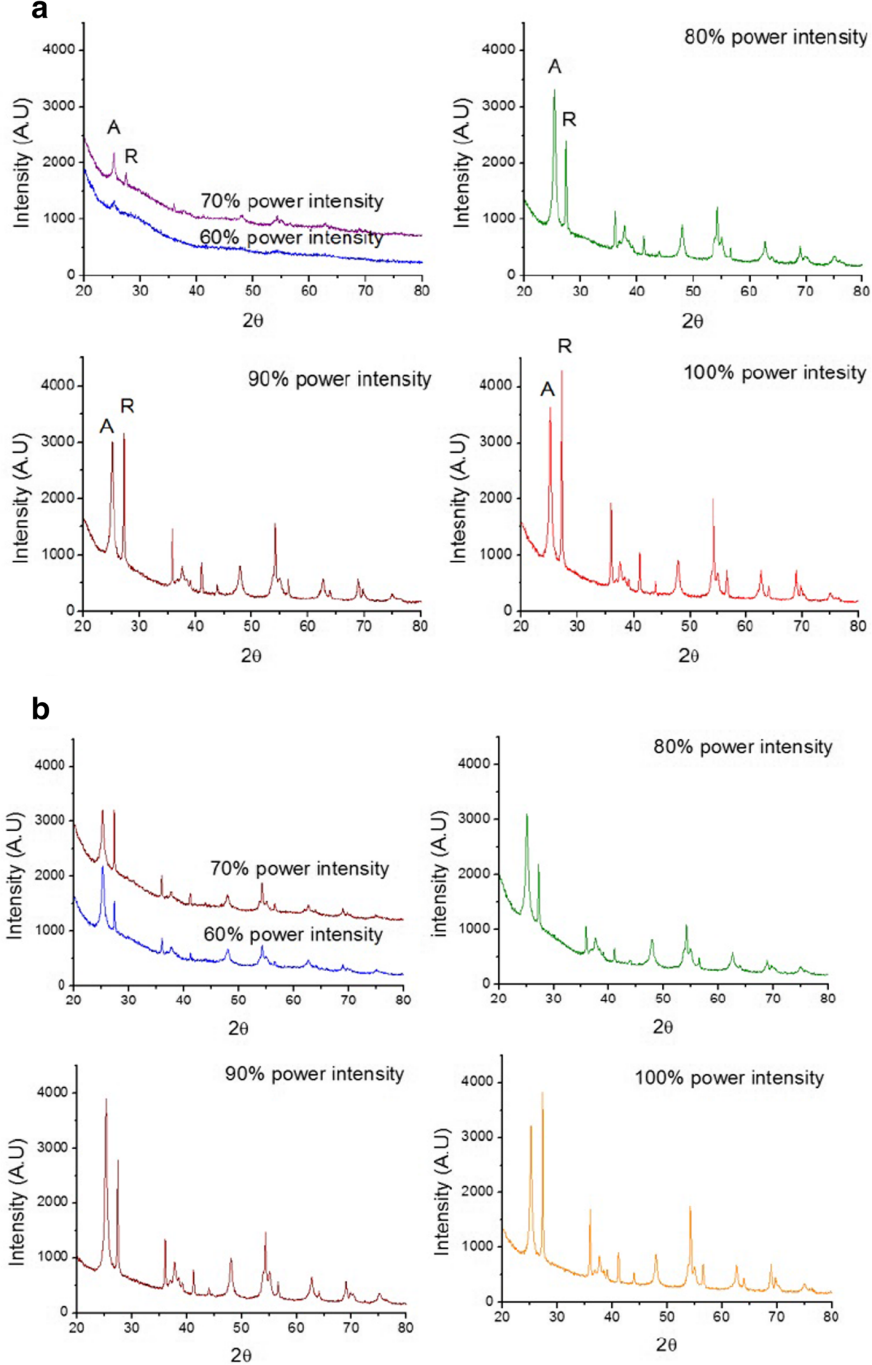
Diffractograms of titanium oxide samples with nitrogen plasma treatment at different intensities of power and treatment times. **a** 60 min. **b** 120 min

Samples of amorphous TiO_2_ powder were treated with nitrogen plasma at different powers for 1 and 2 h. After the treatments with plasma, the XRD analyses (Fig. 4) reveal that xerogel begins to undergo changes in its crystalline structure. At a power of 60% and 1 h of treatment (Fig. 4a), a small peak corresponding to anatase can be observed whose intensity increases when the power is increased to 70%. However, with a treatment time of 120 min and the same intensity of 60%, the growth in parallel with anatase and rutile is observed (Fig. 4b).

Parallel crystallization of anatase and rutile is observed during the process of the plasma treatment; the proportions change as the power of the plasma and treatment time is increased. Unlike the classic heat treatments, plasma treatment allows the crystallization in parallel with anatase and rutile phases whereas its relation depends on the used power and the treatment time (Fig. 4).

The percentage of the anatase in the samples treated with nitrogen plasma and at a temperature of 700 °C for different treatment times was determined using the equation of Spurr [24].

1 $$ {F}_{\mathrm{A}}=\frac{I_{\mathrm{A}}}{I_{\mathrm{A}}+1.265{I}_{\mathrm{R}}}, $$

where *F*_A_ is the fraction of the anatase phase, *I*_A_ is the integral intensity of the anatase phase peak (101), and *I*_R_ is the integral intensity of the rutile phase peak (110). The results are presented in Table 1.

**Table 1:** Comparison of the percentage of anatase with different treatment times, between the samples treated at 700 °C with the applied plasma power

Time (min)	Anatase (%)					
	Temperature (°C)	Power plasma (%)				
	700	60	70	80	90	100
60	82	100	75	72	60	54
120	62	85	75	81	73	52

The Scherrer equation [25] was applied to determine the size of anatase and rutile crystallites in the treated samples.

2 $$ L=\frac{k\lambda }{\beta \cos \theta }, $$

where *β* is the full width at half maximum (FWHM) of the maximum peak, *θ* is the Bragg angle, *λ* = 0.15418 nm, and taking into consideration a factor *k* = 0.94.

As shown in Table 1, the increase in treatment time at a constant temperature of 700 °C reduces the percentage of anatase from 82 up to 62%. However, in the plasma treatment, the variation in the percentage of anatase when the treatment time is higher, it only shows smaller changes in the proportion of anatase. The change in the concentration of anatase is more affected by increasing of the power than by increasing the treatment time. If we consider that the complete crystallization begins at a power of 80% as has been demonstrated in the results of XRD in Fig. 4, the percentage of anatase changes from 72 to 54% and 81 to 52% when the power is increased from 80% up to 100% with treatment times of 60 and 120 min, respectively. The time and the applied power allow for an adequate control of the anatase-rutile proportion.

**Fig. 5:**
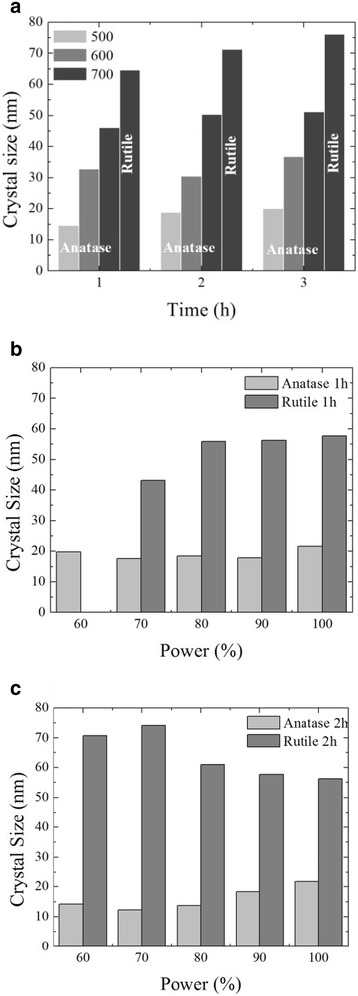
Crystallite size of anatase and rutile for samples treated thermally (**a**), with plasma (**b, c**), at different time intervals

Furthermore, the application of Eq. (2) for both treatments showed a significant growth in crystallite size when the temperature is increased from 500 to 700 °C and the treatment time remains constant, 1, 2, or 3 h. In this way, the size of the anatase crystallite increases from 15 nm up to 46 nm when the temperature increases from 500 to 700 °C in 1 h, with the respective appearance of rutile at 700 °C with a crystallite size of 64 nm. With a treatment time of 3 h, the crystallite size of anatase changes from 20 nm up to 50 nm, with a rutile crystallite size of 76 nm (Fig. 5a). From the results of the equation of Scherrer, along with the diffractograms of Fig. 3, it can be concluded that the optimal treatment to obtain only anatase would be 600 °C with 2 h of treatment, since higher time of treatment, 3 h, increases the crystallite size of anatase (Fig 5a) and also initiates the crystallization of rutile (Fig 3f). On the other hand, a treatment at 700 °C involves the coexistence of two phases (anatase and rutile), with a crystallite size of anatase and rutile that changes from 46 and 64 nm up to 51 and 76 nm, respectively, when the heat treatment is for 1 and 3 h.

The evolution of the size of anatase and rutile crystals under nitrogen plasma treatment at different powers and times from 60 to 120 min reveals minimal changes in the crystallite size. In Fig 5b, it can be seen that the crystallite size for anatase with a treatment time of 60 min at different power remains fairly constant with an approximate value of 20 nm. On the other hand, with a treatment time of 120 min, the size of the crystallites presents small variations within a range of 15–20 nm (Fig. 5c).

Taking into consideration that the crystallization of both phases was obtained with an application of 80% of plasma power intensity (Fig. 4), the size of crystallites remains fairly constant (minimal changes are observed), 15, and 60 nm for anatase and rutile, respectively, with treatment times of 60 and 120 min (Fig 5b, c). Unlike the heat treatment, the increase in plasma power and treatment time has no significant effect on crystallite size, with a more nanostructured material being obtained with the plasma treatment, in comparison with the heat treatment.

**Fig. 6:**
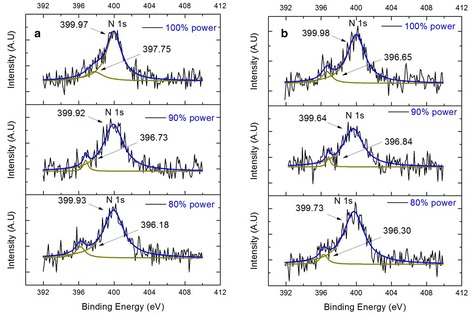
XPS spectra for the titanium oxide samples treated with plasma at different powers (80, 90, and 100%) and treatment times, **a** 60 and **b** 120 min

The plasma treatment presents yet another advantage in comparison with the heat treatment. As has been demonstrated in previous works, the application of nitrogen plasma facilitates the incorporation of nitrogen in the TiO_2_ crystal lattice [18, 19]. In order to determine the presence of nitrogen in the crystal lattice of titanium oxide after the treatments with nitrogen plasma, an analysis of the surface chemical composition and chemical states of the plasma samples were performed using the XPS technique (Fig 6). This figure shows the XPS spectra of N 1s profile with treatment times of 60 and 120 min when the samples are treated with nitrogen plasma at different intensities (80, 90, and 100%). From these studies, two peaks were detected, corresponding to N 1s core level binding energy (BE) ~396 and ~400 eV. Previous studies have reported the appearance of these peaks as a result of the treatment with nitrogen plasma [19]. Analysis of the N 1s peak in the range 396–397 eV has been assigned to β-N atoms in Ti-N bonds. Many researchers have observed N 1s peak around 399–400 eV, corresponding to chemisorbed γ-N_2_ molecules incorporated into TiO_2_ lattice; however, other researchers have assigned these peaks to nitrogen in N-O bond because, at room temperature, molecular N_2_ is not chemisorbed on metal oxides. Both peaks are an evidence of the partial substitution of oxygen by nitrogen atoms, indicative of the N^3−^ species [26] in the crystalline lattice of titanium oxide which favors the chemical bond Ti-N-O. All this is a result of the constant bombing of nitrogen ions by the plasma that generates oxygen vacancies which in turn allows the incorporation of nitrogen atoms in the lattice and thus to obtain, at the end of the process, the compound N_β_TiO_2-X_ (in previous works denominated N-doped-TiO_2-X_, a material which possesses new optical and photocatalytic properties [18, 19]), where *β* is the fraction of nitrogen substituting oxygen in the crystal lattice of titanium oxide when nitrogen atoms partially occupy the oxygen vacancies generated by sputtering effects of the plasma on the TiO_2_, where 2-X>>*β*.

In both graphs, the signal of the N 1s peak in chemical bond Ti-N-O is observed. The incorporation of nitrogen is effected as soon as the phase change is carried out as a result of the plasma treatment, in this case, in a process of parallel growth of anatase and rutile, and when the nitrogen can be incorporated in the structure of anatase and rutile, without affecting the size of the crystal. As can be seen in Fig. 5a, b, crystallite size remains without significant changes when applying a power of 80% or superior.

## Conclusions

Nitrogen plasma treatment for amorphous titanium oxide is a new methodology to obtain anatase and rutile. This process facilitates a rapid and controlled parallel growth of anatase and rutile crystalline phase, resulting in a material with more nanostructured characteristics obtained with less energy consumption, in comparison with heat treatments which require a high consumption of energy due to high temperatures and long treatment times (3 h). The treatment with plasma has shown to be an effective method for obtaining both phases. Moreover, it is demonstrated that nitrogen plasma has the capacity to incorporate nitrogen atoms into the crystal lattice of titanium oxide to obtain N_β_TiO_2-x_ (also known as N-doped TiO_2-x_). Treatments involving the application of plasma to promote phase changes represent a new alternative way of obtaining allotropic forms, such as anatase and rutile for titanium oxide, with low energy costs, and also provide a new treatment option with broad applications, in particular, those that have catalytic applications such as TiO_2_.

## Abbreviations

FWHM: Full width at half maximum
TGA: Thermogravimetric analyses
TiO_2_: Titanium oxide
XPS: X-ray photoelectron spectroscopy
XRD: X-ray diffraction
